# Targeted Deposition of Antibodies on a Multiplex CMOS Microarray and Optimization of a Sensitive Immunoassay Using Electrochemical Detection

**DOI:** 10.1371/journal.pone.0009781

**Published:** 2010-03-19

**Authors:** John Cooper, Nina Yazvenko, Kia Peyvan, Karl Maurer, Chris R. Taitt, Wanda Lyon, David L. Danley

**Affiliations:** 1 CombiMatrix Corporation, Mukilteo, Washington, United States of America; 2 United States Naval Research Laboratory, Washington, D.C., United States of America; 3 Air Force Research Laboratory, Dayton, Ohio, United States of America; Charité-Universitätsmedizin Berlin, Germany

## Abstract

**Background:**

The CombiMatrix ElectraSense® microarray is a highly multiplex, complementary metal oxide semiconductor with 12,544 electrodes that are individually addressable. This platform is commercially available as a custom DNA microarray; and, in this configuration, it has also been used to tether antibodies (Abs) specifically on electrodes using complementary DNA sequences conjugated to the Abs.

**Methodology/Principal Findings:**

An empirical method is described for developing and optimizing immunoassays on the CombiMatrix ElectraSense® microarray based upon targeted deposition of polypyrrole (Ppy) and capture Ab. This process was automated using instrumentation that can selectively apply a potential or current to individual electrodes and also measure current generated at the electrodes by an enzyme-enhanced electrochemical (ECD) reaction. By designating groups of electrodes on the array for different Ppy deposition conditions, we determined that the sensitivity and specificity of a sandwich immunoassay for staphylococcal enterotoxin B (SEB) is influenced by the application of different voltages or currents and the application time. The sandwich immunoassay used a capture Ab adsorbed to the Ppy and a reporter Ab labeled for fluorescence detection or ECD, and results from these methods of detection were different.

**Conclusions/Significance:**

Using Ppy deposition conditions for optimum results, the lower limit of detection for SEB using the ECD assay was between 0.003 and 0.01 pg/ml, which represents an order of magnitude improvement over a conventional enzyme-linked immunosorbant assay. In the absence of understanding the variables and complexities that affect assay performance, this highly multiplexed electrode array provided a rapid, high throughput, and empirical approach for developing a sensitive immunoassay.

## Introduction

The CombiMatrix CustomArray® microarray and ElectraSense microarray are complementary metal oxide semiconductor (CMOS) chips with 12,544 electrodes that can be addressed individually or in user-defined groups. These arrays are available commercially as custom DNA chips with different nucleic acid probe sequences produced at each electrode using sequential electrochemical reactions to add phosphoramidites [1]. Hybridization to probes can be detected using cyanine (Cy) dyes and fluorescent scanners or, alternatively, using horseradish peroxidase (HRP) and enzyme-enhanced electrochemical detection (ECD) on CombiMatrix's microarray readers.

Dill et al [2] first described a method for fixing capture antibodies (Abs) on the 1000-electrode CustomArray microarray, a predecessor of the current ElectraSense microarray. They synthesized different DNA probes on individual electrodes and used Abs tagged with complementary oligonucleotides to self-assemble specifically on individual electrodes of the multiplex array. The array had capture Abs against ricin, *Bacillus globigii* spores, M13 phage, α1 acid glycoprotein, and fluorescein. Initially, antigen (Ag) binding was measured optically, using fluorophore-labeled target or reporter Ab. In later studies [3], [4], the authors used amperometry and HRP with peroxide and ortho-phenylenediamine. They reported that the multiplex microarray and assay demonstrated high specificity and sensitivity in the low pg/ml range.

In more recent studies, we determined that the conjugated Abs were fragile, expensive, and difficult to produce reliably. As an alternative, we investigated using polypyrrole (Ppy) to adsorb Abs to individual electrodes on the array. This compound belongs to a family of conducting polymers that includes polythiophene and polyaniline that have been used to fix proteins and other biomolecules on electrodes for detection using different electrochemical methods. Their use has been well documented in numerous reviews [5], [6], [7], [8], [9], [10], [11], [12], [13], [14], [15], [16]. Ramanaviciene and Ramanavicius [8] singled out Ppy for its biocompatibility, its ability to transduce energy into electrical signals, its protective properties against electrode fouling, and its potential for *in situ* modification.

In this communication, we report on using the CombiMatrix ElectraSense microarray with manual and automated instrumentation for the selective electrochemical deposition of Ppy and adsorption of capture Abs. By designating groups of electrodes on the array for different Ppy deposition conditions, we determined that the use of constant voltage or constant current and the length of time for Ppy deposition influenced the sensitivity and specificity of an immunoassay for staphylococcal enterotoxin B (SEB) as measured using a secondary Ab labeled with Cy5 for fluorescence detection or HRP for ECD. Under optimum conditions, ECD was at least an order of magnitude more sensitive than an ELISA plate immunoassay. In the absence of understanding the variables and complexities that affect assay performance, a highly multiplexed electrode array provides a rapid, high throughput, and empirical approach for developing sensitive immunoassays.

## Results

### Instrumentation

**Figure 1:**
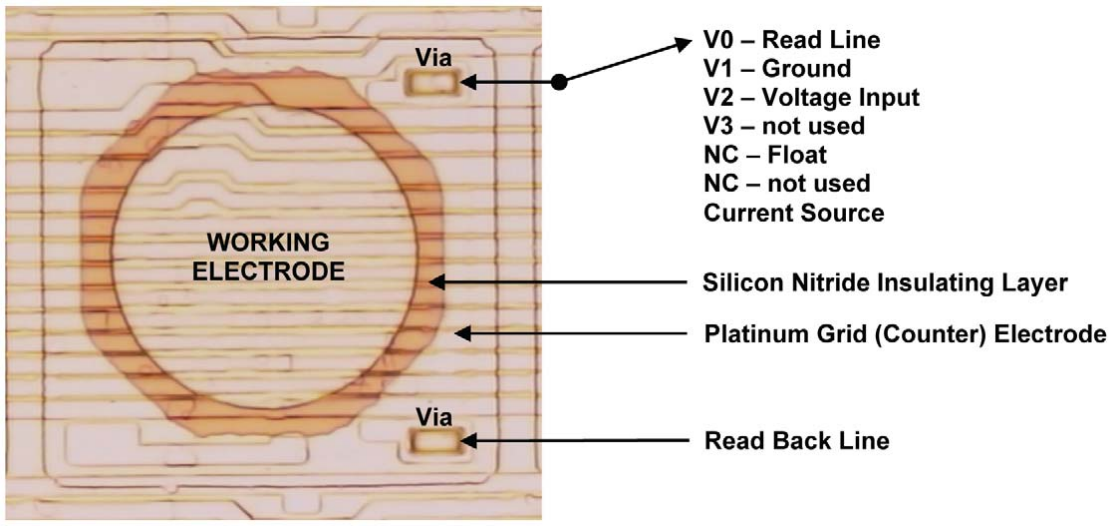
Photomicrograph of a single electrode. The image shows relationships among the different components and their electrical connections.

The ElectraSense microarray, ElectraSense Reader and methodology for ECD have been described previously [17], [18]. Each ElectraSense microarray has 12,544 individually addressable electrodes that are connected by CMOS circuitry. Thirteen pogo pads on the side of the array provide electrical contact with instrumentation to support different transducer functions. Figure 1 shows a photomicrograph of a single electrode on the array. The Pt working electrode is 44 µm in diameter and is separated by a layer of silicon oxynitride from a Pt counter electrode (grid) that is continuous across the surface of the array. The surface of the working electrode is corrugated because of the underlying CMOS circuitry, which connects the electrode to V-lines that create different electrode states.

**Figure 2:**
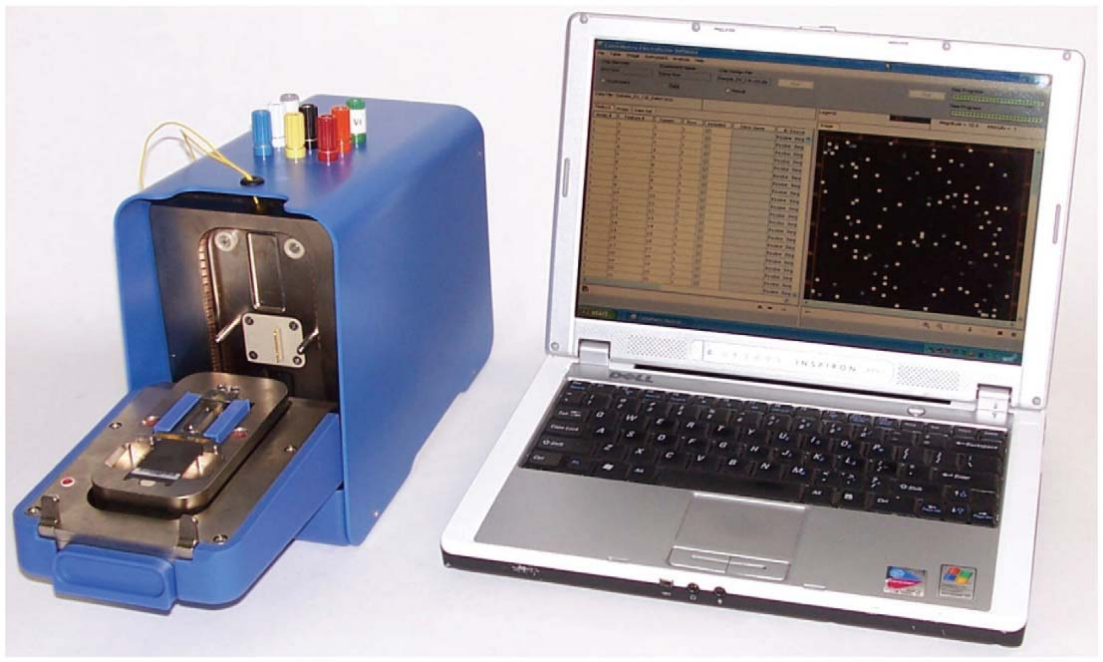
Photograph of the PotentioSense Workstation with computer. This modified ElectraSense reader has externalized leads on the case, which provide connections between electrodes on the array and a potentiostat or other external power source.

Initial studies on the deposition of Ppy and Ab were conducted using the PotentioSense™ Microarray Workstation (Figure 2), which was developed to investigate electrochemical processes on the microarray. The instrument software provides a scripting interface, which enables the user to write a protocol (chip map) that controls whether the instrument addresses electrodes individually or in groups. The state of the electrode(s) can be set to source voltage or current, ground, or disconnected (floating). Similarly, current and voltage can be read from a single electrode or a defined group of electrodes using the software and electronics in the instrument and on the microarray. Direct connections to the microarray are externalized on the PotentioSense so that it will interface with third party instruments; e.g., potentiostat, oscilloscope, wave generator, etc. High tolerance electronics are used in the PotentioSense along with software and hardware feedback routines to generate and measure electrical signals accurately. In addition, the instrument is factory calibrated, and calibration values are saved in the device to ensure accuracy and precision.

**Figure 3:**
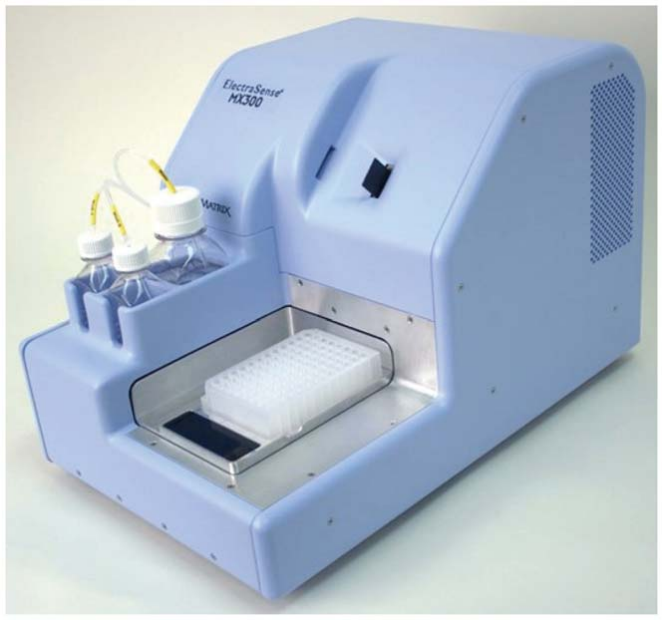
Photograph of the MX300 Automated Microarray Workstation. Reagents are loaded in a 96-well plate, and a programming script is used to control their flow into a single chamber or into four chambers containing an ElectraSense microarray. Current or voltage can be applied to single or groups of electrodes for Ppy deposition, and current can be read from a single electrode or groups of electrodes for ECD.

The MX300 (Figure 3) is an automated fluidic handling and electrochemical processing station for the ElectraSense microarray that includes an automated fluidic handling system and all of the electronics and software found on the PotentioSense. Using a standard 96-well plate, the user can load any combination of reagents and direct their introduction onto the microarray using the scripting program. This instrument can deposit Ppy and different Abs on different electrodes; and, using a different set of instructions and reagents, it can run an ECD immunoassay to determine Ag concentration in one or more samples. For Ppy deposition, the MX-300 was configured with a single chamber that covered the array (12K configuration). For antigen detection, the MX-300 was configured with four separate chambers (4×2K configuration) with 2,000 electrodes in each chamber. This allowed analysis of multiple samples on a single microarray.

### Initial Demonstration of Antibody Attachment to Individual Electrodes

**Figure 4:**
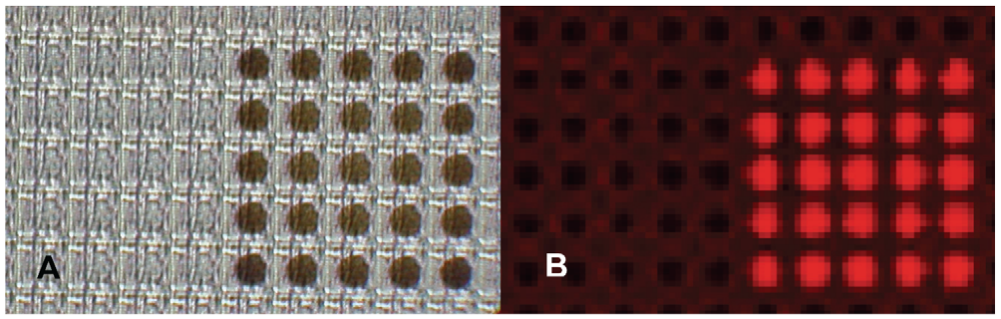
Deposition of Ppy and Ab on individual electrodes. A) Photomicrograph of an ElectraSense microarray under epi illumination and showing the deposition of polypyrrole with adsorbed murine IgG on a 5×5 set of electrodes. B) Fluorescence scanned image of the microarray treated with Cy5-labeled goat anti mouse IgG and showing the presence of murine Ab on the electrodes.

A number of approaches were investigated to develop a microarray immunoassay that would improve upon the method of using nucleic acid-Ab conjugates as capture elements. Initial studies involved spotting Abs on the array; however, spotting created uneven depositions across numerous electrodes, which caused uneven and variable fluorescent and ECD measurements. Subsequent efforts focused on using electropolymerized Ppy deposition to entrap or adsorb antibodies on individual electrodes. For each experiment, a chip map was created that directed the application of a set potential to groups of 5×5 electrodes on the array, Ppy was electrodeposited applying 1.0 V for 5 s, and murine IgG was selectively adsorbed to the deposited Ppy for 5 min. Figure 4 shows two images of the 5×5 sectors with and without Ppy and Ab. In the light micrograph, Ppy deposition is clearly present as brown spots isolated on each electrode. The array was treated with Cy5-labeled goat anti-mouse IgG, and the fluorescence image shows that the antibodies were localized only on electrodes with Ppy.

**Figure 5:**
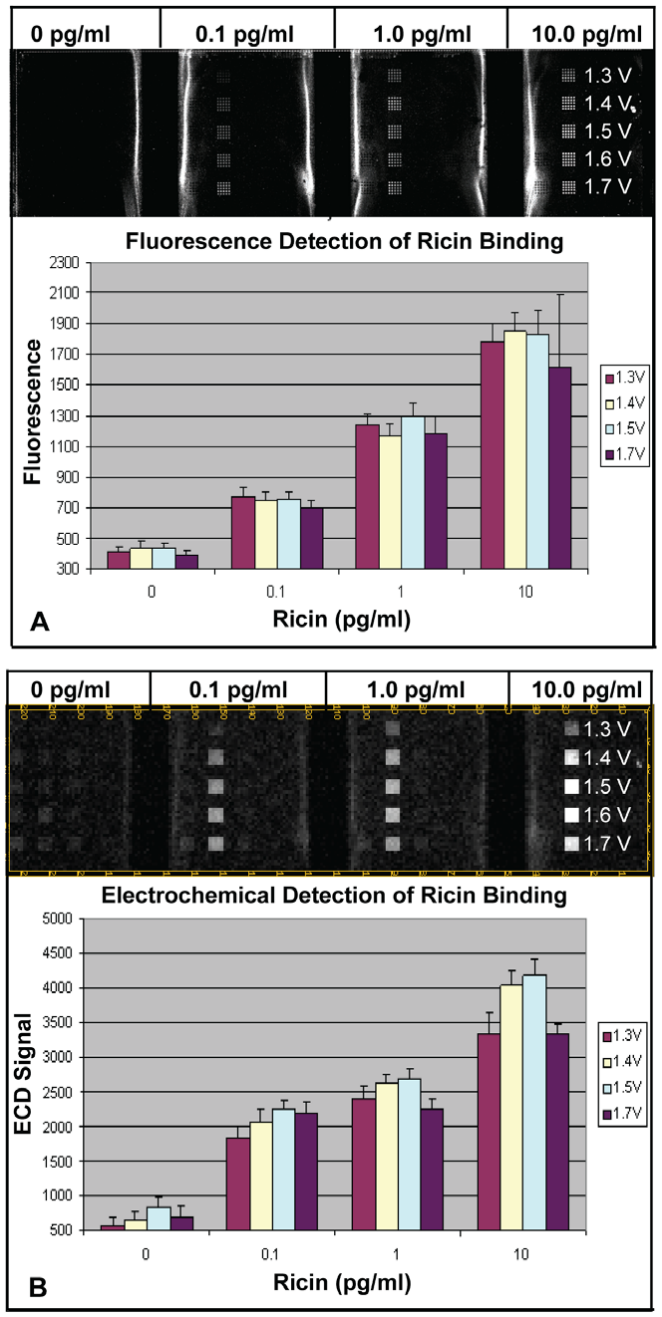
Detection of ricin binding to murine anti-ricin MAb adsorbed on Ppy. Polypyrrole was deposited at different voltages followed by adsorption of anti-ricin MAb. Three concentrations of ricin were incubated for 1 h in different chambers of a four-chamber hyb cap. Binding was detected using biotin-labeled goat anti-ricin Ab. A) Results using Cy5-SA showing a scanned fluorescence image of the array and a graph illustrating fluorescence intensities for different groups of electrodes. B) Results using HRP-SA showing a pseudo image of the array and a graph illustrating the ECD signals for different groups of electrodes.

To determine whether adsorbed Abs on the array were functional, Ppy was deposited in four 5×5 blocks of electrodes at different voltages (1.3, 1.4, 1.5, and 1.7 V) for 5 s; and anti-ricin monoclonal Ab (MAb) was adsorbed onto the electrodes. Three concentrations of ricin (0.1, 1.0, and 10.0 pg/ml) were tested, and binding was detected using biotinylated goat anti-ricin Ab and HRP-streptavidin (HRP-SA) for ECD. After measurement, the array was washed, and Cy5-streptavidin (Cy5-SA) was applied to the same array for fluorescent detection. Figure 5A and 5B illustrate respectively the microarray fluorescence image and the ECD pseudo image that were generated. Bar graphs beneath each image demonstrate that ricin could be detected at 0.1 pg/ml using ECD or fluorescence detection under optimized conditions. In this experiment, increasing the deposition potential to 1.7 V reduced the ECD signals for all concentrations of ricin compared to results using lower deposition voltages; however, this trend was not observed using fluorescence detection.

### Optimization of Antibody Deposition

**Figure 6:**
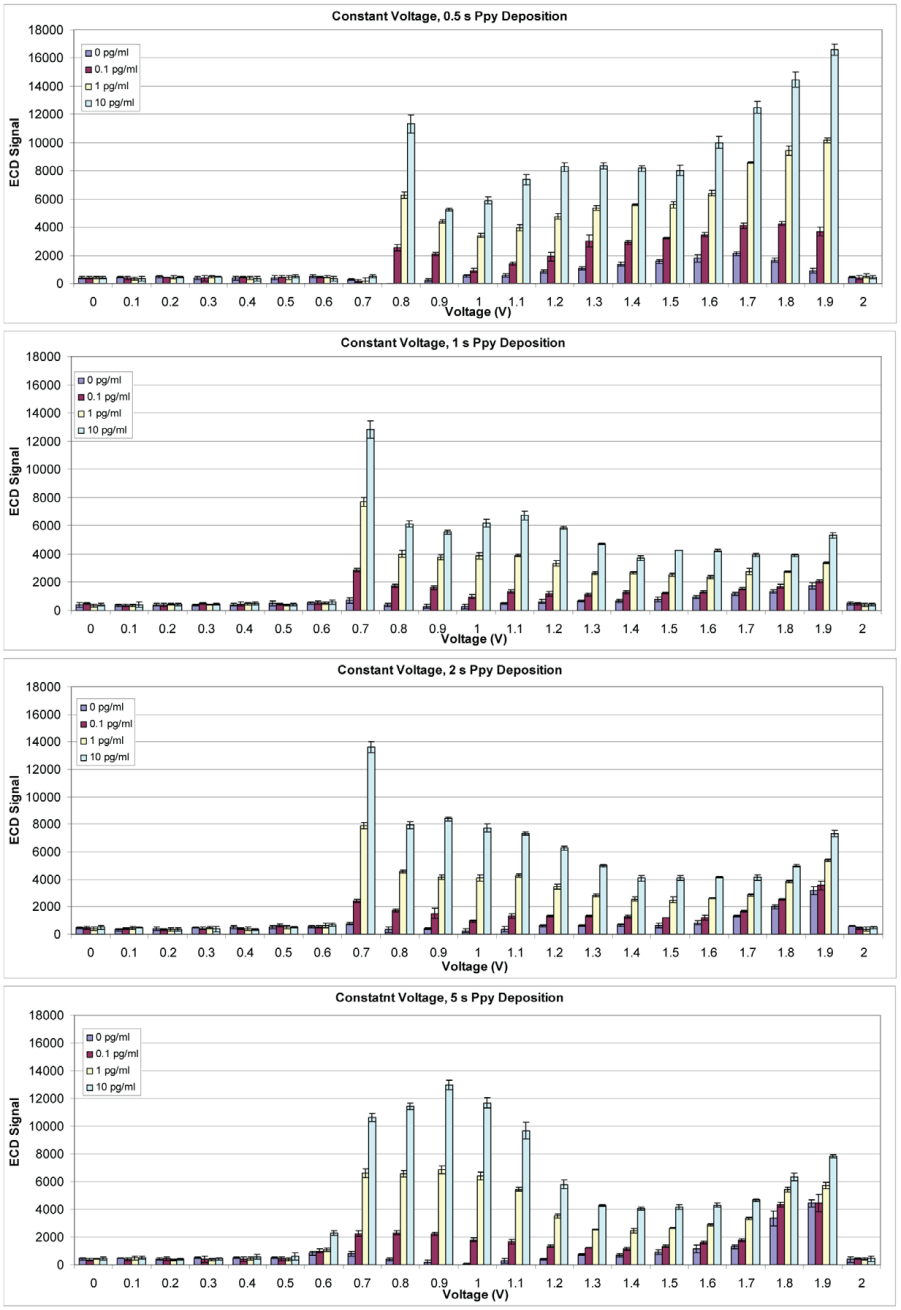
Electrochemical detection of SEB binding on an array with Ppy deposited using constant voltage. Polypyrrole was deposited using potentials from 0.0 to 2 V for 0.5, 1.0, 2.0, or 5.0 s followed by adsorption of anti-SEB MAb. Different concentrations (0.0, 0.1, 1.0 or 10.0 pg/ml) of SEB were incubated in individual chambers of a four-chamber hyb cap, and binding was detected using biotinylated rabbit anti-SEB with HRP-SA.

To explore the relationship between deposition potential and assay sensitivity, a revised assay was developed using SEB as the target. A chip map was written on the MX300 instrument to create blocks of 2×2 electrodes in four sectors that align with a four-chambered (hyb) cap. Each block had a different set of Ppy deposition conditions based upon voltage (0–2 V in increments of 0.1 V) and time during which voltage was applied (0.5, 1.0, 2.0, and 5.0 sec). Anti-SEB MAb was adsorbed to all blocks of electrodes except for a row of control blocks, which were treated with casein only and served as negative controls. Different concentrations of SEB (none, 0.1 pg/ml, 1.0 pg/ml, and 10.0 pg/ml) were tested on the array followed by incubation with biotinylated rabbit anti-SEB Ab and SA-HRP. Figure 6 illustrates that the detection of SEB binding occurred in a defined window of Ppy deposition voltages–0.8 to 1.9 V for a 0.5 s deposition and 0.7 to 1.9 V for 1, 2, and 5 s depositions. Within these windows, deposition voltage influenced non specific binding to electrodes with the lowest values observed using lower voltages (0.9 to 1.0 V) for a 5 s. Higher voltages reduced specific binding and increased non-specific binding.

**Figure 7:**
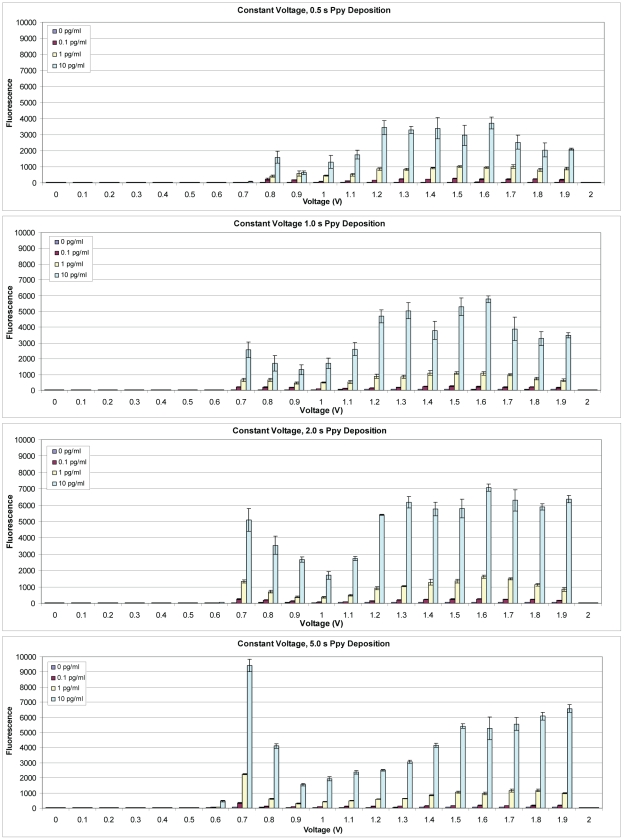
Fluorescence detection of SEB binding on an array with Ppy deposited using constant voltage. Polypyrrole was deposited using potentials from 0.0 to 2 V for 0.5, 1.0, 2.0, or 5.0 s followed by adsorption of anti-SEB MAb. Different concentrations (0.0, 0.1, 1.0 or 10.0 pg/ml) of SEB were incubated in individual chambers of a four-chamber hyb cap, and binding was detected using biotinylated rabbit anti-SEB with Cy5-SA.

**Figure 8:**
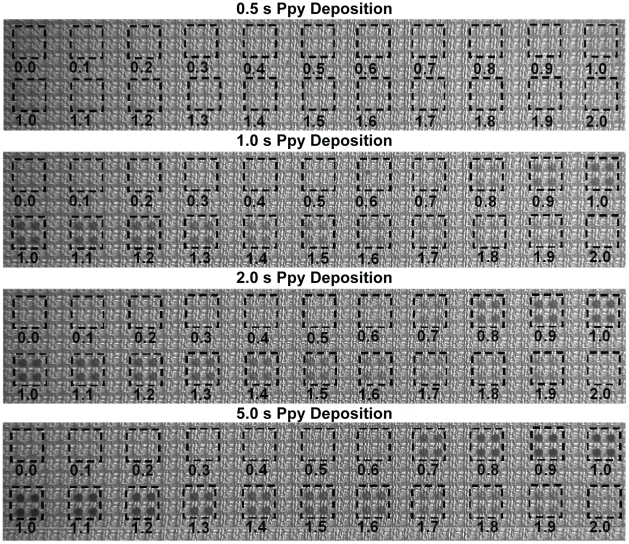
Composite photomicrograph showing Ppy deposition on 2×2 groups of electrodes using constant voltage. Polypyrrole was deposited for 1.0 s using voltages from 0.0 to 2.0 V in 0.1 V increments, as listed beneath each group.

Because the SEB detection was apparent only when voltages between 0.7 and 1.9 V were applied, the assay was run using Cy5-SA and fluorescence detection to determine if this window was related to the Ab deposited on the Ppy or some electrical properties of the Ppy. Figure 7 illustrates that, as observed with ECD, SEB was only detected fluorescently on Ppy deposited between 0.7 and 1.9 V. Within this window of deposition, the fluorescence pattern reflecting assay sensitivity was bimodal like the ECD assay, but non-specific binding to electrodes treated only with casein (control) was very low (data not shown). To understand these patterns better, photomicrographs were made of Ppy deposited on the array. Figure 8 shows the colored pattern of Ppy deposited on blocks of electrodes for 2 s at 0.0 to 2.0 V. Colored product was first apparent at 0.7 V, the intensity of the color appeared to increase with increasing potential to 1.0 V and it then declined thereafter, but was still apparent at 1.9 V.

**Figure 9:**
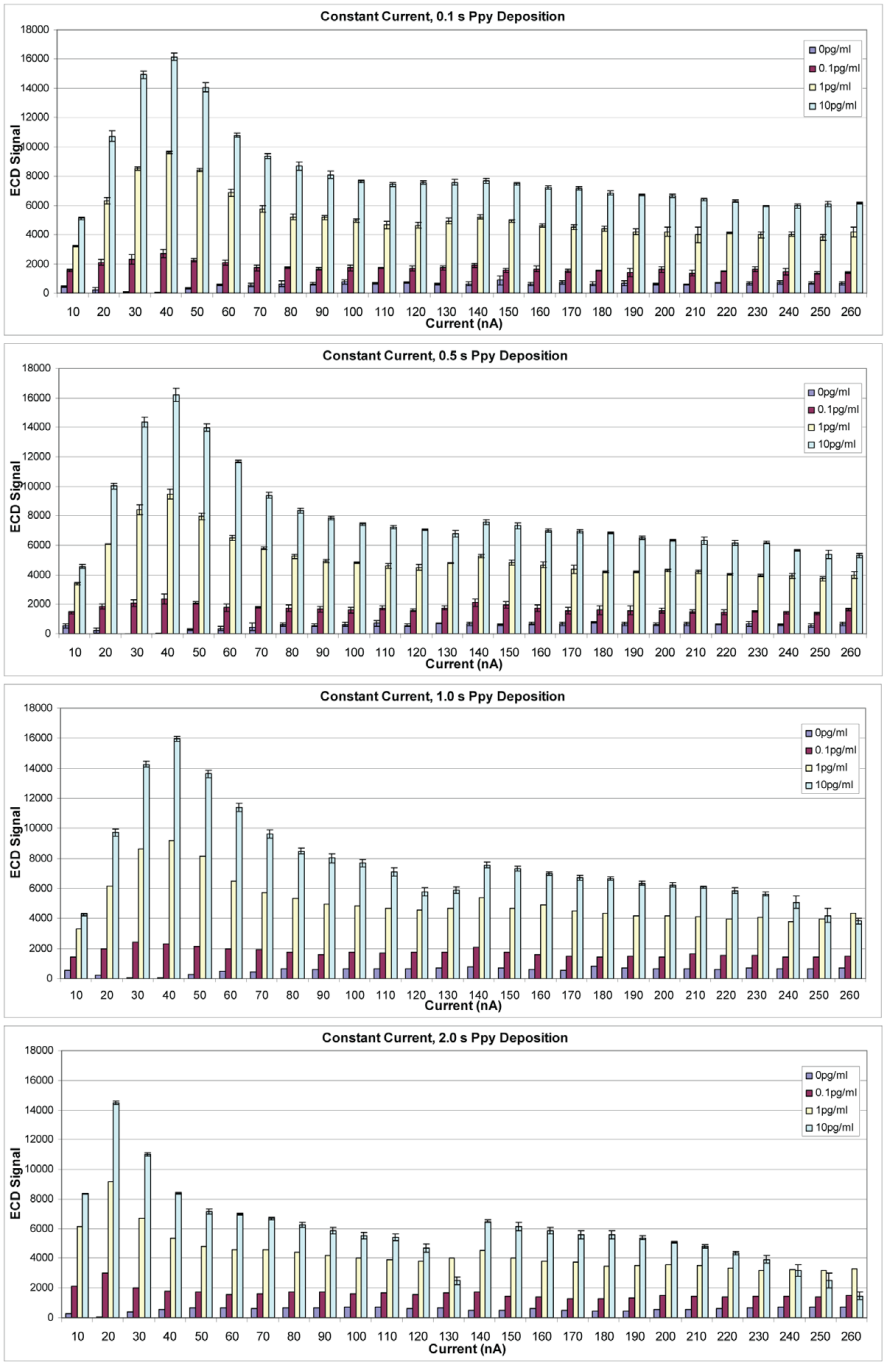
Electrochemical detection of SEB binding on an array with Ppy deposited using constant current. Polypyrrole was deposited using currents from 10 to 260 nA for 0.1, 0.5, 1.0, or 2.0 s. Different concentrations (0, 0.1, 1.0 or 10.0 pg/ml) of SEB were incubated in individual chambers of a four-chamber hyb cap, and binding was detected using biotinylated rabbit anti-SEB with HRP-SA.

**Figure 10:**
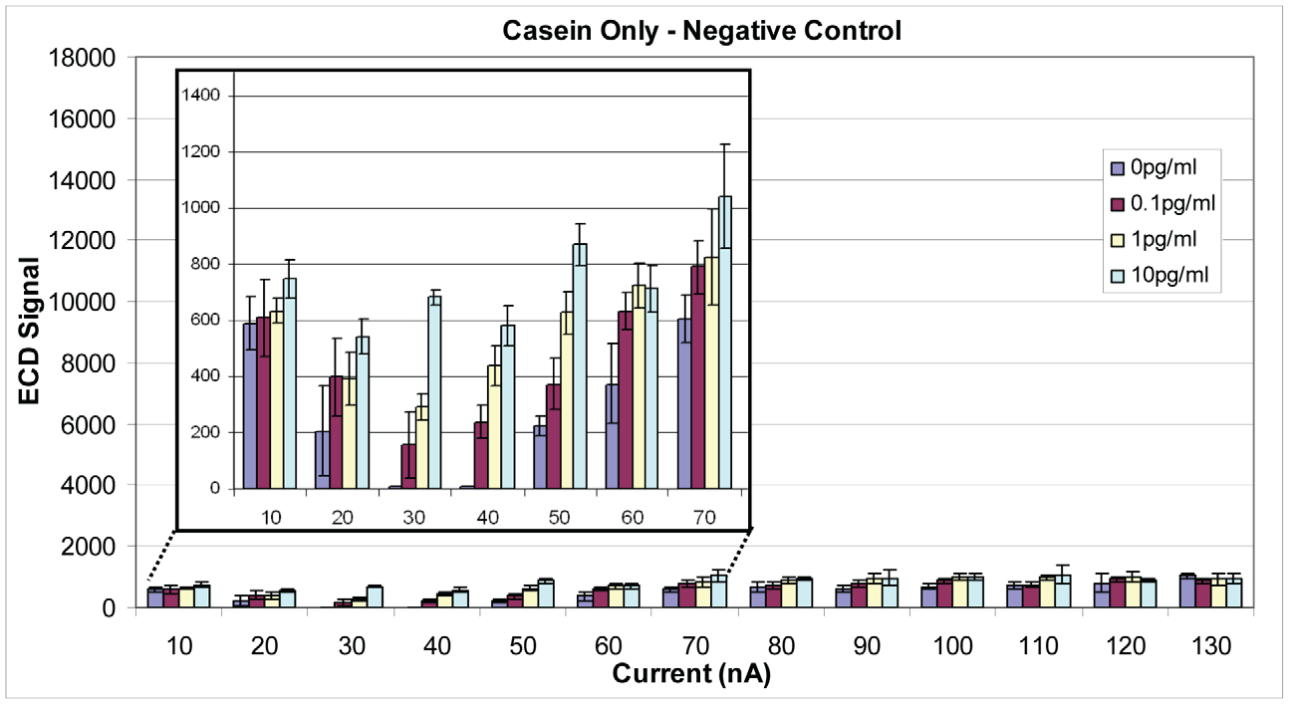
Electrochemical detection of non-specific binding by SEB. Polypyrrole was deposited for 1 s at increasing currents from 10 to 130 nA and blocked with saturated casein in place of capture Ab. Binding was detected using biotinylated rabbit anti-SEB with HRP-SA. Inset graph illustrates that the lowest non-specific binding was obtained using deposition currents of 30 and 40 nA.

**Figure 11:**
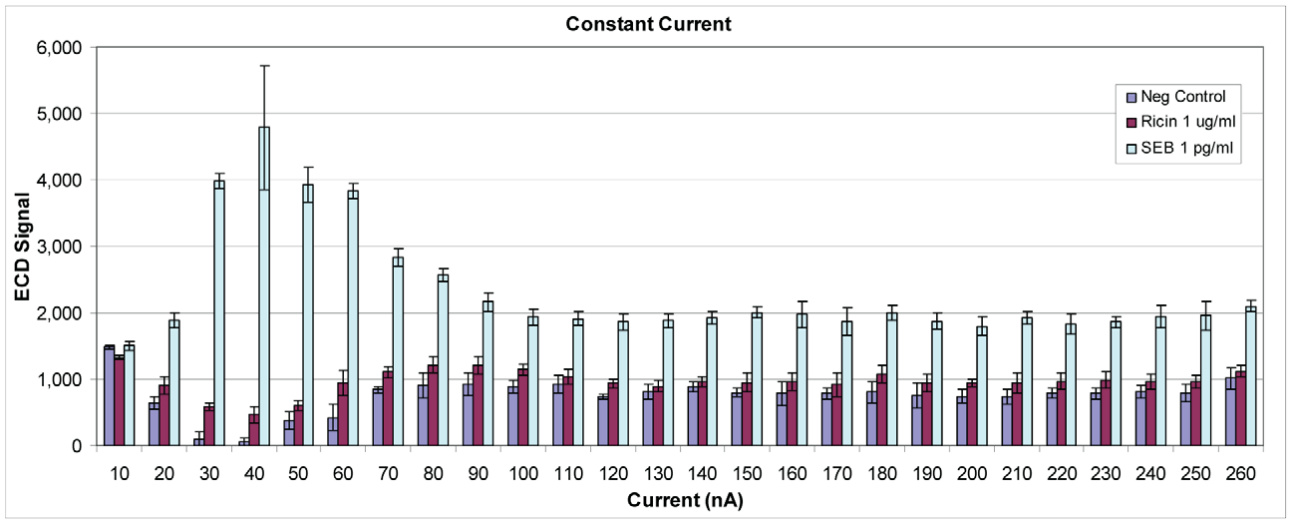
Electrochemical detection of cross reactivity by excess ricin with rabbit anti-SEB capture Ab. Polypyrrole was deposited for 1 sec at increasing currents from 10 to 260 nA. Biotinylated rabbit anti-SEB Ab was used to detect SEB and biotinylated goat anti-ricin Ab was used to detect ricin. The lowest non-specific binding and best sensitivity (Ag versus no Ag) was obtained using deposition currents of 30 and 40 nA.

As an alternative to constant voltage, Ppy was deposited using constant current. Assay conditions were identical to those described above, except for deposition times (0.1, 0.5, 1.0 and 2.0 s) and current. Figure 9 illustrates results from an assay where Ppy was deposited using 10–260 nA. The best sensitivity, as measured by ECD, was obtained when a deposition current of 40 nA was applied for 0.1, 0.5, or 1.0 s. When current was applied for 2.0 s, the curve moved to the left, and the best activity was observed with a lower deposition current of 20 nA. Polypyrrole was deposited using a broader range of currents (0–980 nA) for 1 s, and ECD signals increased to a peak at 60 nA and then declined with increasing deposition currents (data not shown). Figure 10 illustrates SEB binding to negative control electrodes that lacked capture Ab and were blocked with casein. The lowest non-specific binding to the electrodes was observed when Ppy was deposited at 20–40 nA for 1 s. At currents below and above those values, non-specific binding increased both in the presence and absence of SEB suggesting binding by other assay reagents (e.g., biotinylated secondary Ab and SA-HRP). Figure 11 illustrates cross reactivity of the anti-SEB array in the presence of 10^6^-fold excess of ricin. As observed in the previous experiment, ricin binding was minimal when the Ppy was deposited at 30–40 nA for 1 s but increased on Ppy deposited at lower and higher currents. That ricin binding increased along with the negative control (no Ag), which supports the idea that other assay reagents contributed to the non-specific signal.

**Figure 12:**
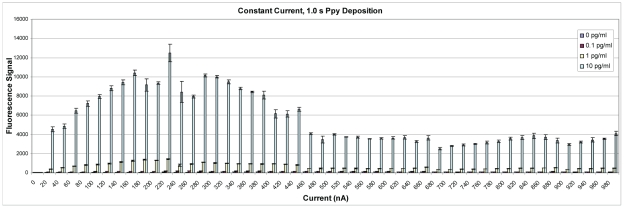
Fluorescence detection of SEB binding on an array with Ppy deposited using constant current. Polypyrrole was deposited using constant current from 0 to 980 nA for 1.0 s. Different concentrations (0, 0.1, 1.0, and 10.0 pg/ml) of SEB were incubated in individual chambers of a four-chamber hyb cap, and binding was detected using biotinylated rabbit anti-SEB with Cy5-SA.

**Figure 13:**
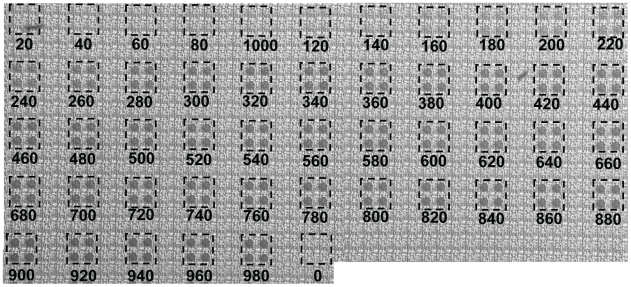
Composite photomicrograph showing the deposition of Ppy on 2×2 groups of electrodes. Polypyrrole was deposited using constant current (0.0 to 980 nA) for 1.0 s as listed beneath each group.

The constant current ECD assay was repeated using fluorescence detection, and Figure 12 shows that the fluorescence signal improved as the Ppy deposition current increased up to 220 nA after which signal decreased with increasing deposition currents. Non-specific binding to electrodes treated only with casein (control) was minimal throughout the range of currents used for Ppy deposition (data not shown). The pattern of colored Ppy was examined microscopically, and Figure 13 shows that colored product was apparent on electrodes after a 1 s deposition at 160 nA. The intensity of the colored product increased and appeared to reach a plateau thereafter, but did not demonstrate the loss of color intensity that was observed with constant voltage deposition.

### Comparison of Assay Performance

**Figure 14:**
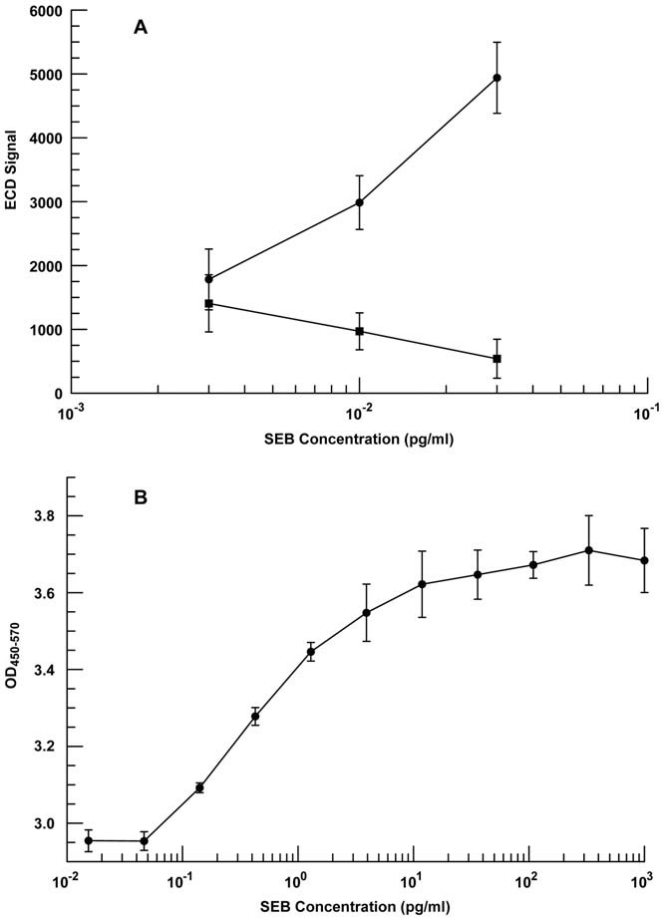
Determination of the LOD for SEB detection. **A**) Three concentrations (0.003, 0.01, and 0.03 pg/ml) were incubated on a microarray with either anti-SEB MAb (•-•) or casein (▪-▪) adsorbed onto Ppy that was deposited at 40 nA for 1 s. SEB binding was detected using biotinylated rabbit anti-SEB Ab and SA-HRP. Background (0 pg/ml SEB) was 1147±283. **B**) Different concentrations of SEB were incubated on a microtiter plate with anti-SEB MAb adsorbed onto the surface of each well. SEB binding was detected using biotinylated rabbit anti-SEB Ab and SA-HRP. Background (0 pg/ml SEB) was OD_450–570_ 2.939±.026.

To determine a lower limit of detection (LOD) for the enzyme-enhanced ECD assay, lower concentrations of SEB were tested using a new version of the manual ElectraSense reader with improved electronics that reduce electronic noise and increase ECD signals. Figure 14A shows that the assay was able to detect 0.01 pg/ml in buffer but not at 0.003 pg/ml. A standard ELISA microwell plate assay was developed around the same capture and secondary Abs, and Figure 14B illustrates that this assay detected SEB at 0.15 pg/ml but not at 0.05 pg/ml, which indicates that the ECD assay was at least an order of magnitude more sensitive than the ELISA plate assay.

## Discussion

This communication demonstrates the use of a commercial, highly multiplex CMOS microarray for the automated deposition of Ppy and adsorption of capture Abs for detection of antigen binding using ECD or fluorescence detection. To support multiple assays and high throughput on the array, a four-chamber hyb cap was used so that four different concentrations of Ag could be applied on a single array; and, within each chamber, Ppy deposition was controlled on 50 blocks of 4 electrodes with respect to deposition time and voltage or current. In this configuration, a single microarray supported 200 different experimental conditions with respect to Ppy deposition and Ag concentrations. Moreover, Ag binding could be studied using ECD or fluorescence detection. The ability to perform such a large number of experiments in parallel demonstrates the power of this methodology. Attempting these studies using a single Pt electrode mounted in an electrochemical cell might provide good control over electrochemical processes but would lack throughput and versatility.

Our studies initially used constant voltage to electropolymerize the Ppy, which others have used with a two compartment electrochemical cell where a reference electrode can maintain the applied voltage. Ramanavicius et al (15) reported that using their potential pulse technique with a range of 0.6 to 1.2 V versus Ag/AgCl for initial structuring of the Ppy was most suitable for entrapping biologically active materials. These values correspond closely to our findings that SEB detection was observed only when the Ppy was deposited between 0.7 and 1.9 V.

While constant voltage was used successfully for electropolymerizing pyrrole, deposition voltages are affected by the number of electrodes addressed and were difficult to maintain using the MX300 in the absence of a reference electrode. However, using this instrument with constant current provided consistent, automated deposition of Ppy and capture Ab because currents could be maintained at designated electrode regardless of the number of electrodes addressed. Our results show that excellent SEB detection using ECD could be achieved by applying a low current (30–50 nA) for a very brief period of time (1 s). Using fluorescence detection, the SEB assay performed well over even broader range of Ppy deposition currents.

Sadki et al [19] reviewed the physical, electrical and chemical parameters that influence the electropolymerization of pyrrole and identified monomer substitution, electrolyte (dopant), solvent, pH, electrochemical method, and temperature as influencing the formation and characteristics of a Ppy film. From a practical standpoint of developing an optimized immunoassay using Ppy, these parameters can be studied efficiently and effectively using the ElectraSense microarray and either the PotentioSense or MX300 instruments. This study demonstrates that assay sensitivities can vary considerably with Ppy deposition time and voltage or current. Moreover, non-specific binding to the Ppy also appears to vary with deposition conditions. Those that produce optimal ECD sensitivities may differ from those providing optimal fluorescence detection.

Photomicrographs of Ppy deposition on electrodes illustrate additional factors that can influence the performance of ECD and fluorescent assays. With constant voltage deposition, appearance of colored Ppy spots roughly corresponded to the results of the ECD and fluorescent assays with detection occurring on Ppy deposited between 0.7 and 1.9 V. However, with constant current, the best ECD results occurred on Ppy deposited at low current for short periods of time and were colorless on the micrographs, whereas the best fluorescent results peaked at higher currents (220 nA) where colored Ppy began to appear. Although no efforts were made to identify the source of these differences, we conjecture that the two methods of detection are being influenced differently by the Ppy layer. For instance, thin films of electroactive Ppy should facilitate ECD by supporting electron transfer. At the same time, fluorescence detection may be reduced in thinner films because of fluorescence quenching by the Pt. Alternatively, more densely colored films of Ppy could also quench fluorescence, while a thicker, more oxidized Ppy layer might provide more resistance to ECD.

With respect to the performance of the microarray as a platform for detecting SEB, the LOD for ECD assay was between 0.003 and 0.01 pg/ml under optimum conditions and no interferants. This was at least an order of magnitude better than that observed using a standard microtiter plate ELISA with the same Ab reagents. Staphylococcal enterotoxin B is a potent toxin and has been studied extensively because of its association with foodborne illnesses and use as a biological threat agent. Labib et al [20] listed the different immunoassays that have been developed to detect SEB and their LODs, which ranged from ∼0.1 fg/ml to ∼2.5 µg/ml. Results from the ECD assay are at the lower end of detection for the assays listed in their publication. The excellent performance of the ECD assay is related to using the microarray to identify Ppy deposition conditions that maximized the signal from SEB binding while minimizing the signal from non-specific binding.

While distinct in the approach, optimizing Ppy deposition appears similar to, but more exact, than treatment of polystyrene to produce high protein binding surfaces on beads and plates. The commercial instruments and CMOS microarray described in this communication offer a broad set of tools for developing protein assays on a compact, high throughput platform that supports both electrochemical and fluorescence detection. In conjunction with the MX300 instrument, Ppy can be deposited on the microarray under optimal conditions for protein adsorption; and multiplex assays can be developed by sequentially depositing Ppy and different proteins. Finally, Ppy and protein deposition on electrodes is uniform, and groups of electrodes can be used for each capture protein to provide statistical significance. From a practical standpoint of developing a sensitive and specific assay on a Pt electrode, the microarray can rapidly sort these factors empirically to optimize assay results.

## Materials and Methods

### Reagents

For development of the SEB immunoassay, the antigen and antibodies (rabbit anti-SEB and anti-SEB MAb) were purchased from the Critical Reagent Program (Critical Reagent Program, Aberdeen Proving Ground, MD). Ricin was purchased from Sigma-Aldrich (Sigma-Aldrich, St. Louis, MO), and ricin MAb and goat anti-ricin Ab were purchased from the Critical Reagent Program as a secondary (reporter) Ab. The SEB and ricin antibody pairs were evaluated for their functionality as capture and secondary antibodies, and the best results were obtained using the MAb as the capture Ab and the polyclonal Ab as reporters. Both secondary Abs were labeled with EZ-link Sulfo-NHS-LC-Biotin (Thermo Fisher Scientific, Rockford, IL). The protein blocking solution (PBSC) was prepared by mixing three grams of casein (Casein from Bovine Milk, Technical Grade, Sigma-Aldrich) in one liter of phosphate buffered saline (PBS, pH 7.2) with stirring for 1–2 h. The suspension was refrigerated overnight and allowed to filter under gravity flow through a 0.22 µm filter (Steritop-GP, Millipore, Billerica, MA) at 4°C for 24 h. Pyrrole (Sigma-Aldrich) was distilled and stored under argon in sealed glass ampoules at 4°C and protected from the light. The 0.1 M working solution of pyrrole was prepared by diluting the distilled reagent in 0.1 M dibasic sodium sulfate (Sigma-Aldrich) in water immediately prior to use. Photomicrographs of Ppy deposition on the microarray were made using an Olympus BX60 microscope with epi illumination (Center Valley, PA).

### Deposition of Polypyrrole and Capture Antibody

To deposit the anti-SEB MAb on individual electrodes, a chip map was created for the PotentioSense instrument by designating through the software which electrodes were to be addressed, the voltage or current to be applied, and the time of application. The chip map created four replicated areas on the array that corresponded to the four chambers of a plastic hyb cap (ElectraSense Hybridization Cap, 4×2K, CombiMatrix Corp., Mukilteo, WA). Within each area, 2×2 or 5×5 blocks of electrodes were connected through CMOS transistor switches on the array so that they received the same voltage or current for the same period of time. For manual deposition, a single-chambered hyb cap (ElectraSense Hybridization Cap, 12K) was mounted on the array using a clamp (CustomArray Clamp for 4×2 & 12K) that fits into the PotentioSense. For automated processing, an MX300 with a single chamber (12K configuration) was used. To prevent non-specific binding, the array was treated with PBSC for 5 min, washed three times with PBS containing 0.1% Tween 20 (PBST), three times with PBS, and three times with 0.1 M dibasic sodium sulfate prior to adding pyrrole for electrodeposition. After Ppy deposition, the array was washed twice with PBS; and capture Ab, diluted in PBS, was added for 15 min at 25°C. The array was washed three times with PBSC and blocked with the same for 2–5 mins. After Ab deposition, the microarray was blocked with PBSC for 1 h, treated with Post Coating Buffer (ALerCHEK, Portland, ME), spin coated, and stored at 4°C.

### Microarray Immunoassay

Microarray immunoassays were done manually so that results from experiments using ECD and fluorescence detection were processed in the same manner. For assay, the microarray was fitted with a four-chamber hyb cap and washed with PBSC before 40 µl of Ag in PBSC or PBSC alone (control) were loaded into each chamber. Following a 1 h incubation at 25°C, the chambers were washed five times with PBSC; and biotin-labeled secondary Ab (diluted to 2 µg/ml in PBSC) was added for a 1 h incubation at 25°C. After washing three times with PBSC, the four-chambered hyb cap was removed and replaced with a single-chambered hyb cap, and the array was washed three more times. For fluorescence detection, Cy5-streptavidin (GE Healthcare, Amersham Biosciences, Piscataway, NJ) was added for 1 h, washed five times in PBSC and twice in PBS and scanned on a GenePix 4000B (Axon Instruments, Molecular Devices, Sunnyvale, CA). For ECD, microarrays were incubated for 30 min with Poly-80-HRP Streptavidin (Fitzgerald Industries International, Acton, MA) diluted 1∶1000 in PBSC. Arrays were washed four times with PBSC, once with PBS, and twice with pH 4 Conductivity Buffer Substrate (BioFX, Owings Mills, MD). TMB Conductivity 1 Component HRP Microwell Substrate (BioFX) was added to the array, and it was scanned immediately with an ElectraSense microarray reader (CombiMatrix). Data were quantified using Microarray Imager or ElectraSense software (CombiMatrix) for fluorescent scans or ECD respectively.

### Microwell ELISA

Anti-SEB MAb was diluted 1∶500 in 0.5 M sodium carbonate-bicarbonate pH 9.6 buffer (Sigma) and 25 µl of the solution were added to each well of a 96-well plate (NUNC Immuno MicroWell 96-Well Plate, Thermo Fisher Scientific). The plate was covered and incubated at 4°C over night. Each well was washed five times with 200 µl of PBS with 0.1%Tween 20 (PBST) and blocked with 1X ELISA Diluent Solution (eBioscience, San Diego, CA) for 2 h at 25°C with agitation. An SEB solution (1000 pg/ml) was prepared in 1X Diluent Solution and serially diluted 1∶3 to a lowest concentration of 0.015 pg/ml. Each concentration was added to three wells, and the plate was incubated 1 h at 25°C with agitation. After five washes with PBST, each well received 50 µl of biotinylated rabbit anti-SEB Ab, diluted 1∶1000 in 1x Assay Diluent; and the plate was incubated overnight at 4°C. For detection, the plate was washed five times with PBST; 100 µl of 1xTMB Substrate Solution (eBioscience) were added; and the plate was incubated at 25°C for 15 min with agitation. After this time, 50 µl of Stop Solution (eBioScience) were added to all wells; and the plate was read at 450 nm and 570 nm on a SPECTRAmax PLUS 384 microplate reader (Molecular Devices, Sunnyvale, CA). For data analysis the OD_570 nm_ was subtracted from the OD_450 nm_.
